# *Ixodes scapularis* saliva mitigates inflammatory cytokine secretion during *Anaplasma phagocytophilum* stimulation of immune cells

**DOI:** 10.1186/1756-3305-5-229

**Published:** 2012-10-10

**Authors:** Gang Chen, Maiara S Severo, Mohammad Sohail, Olivia S Sakhon, Stephen K Wikel, Michail Kotsyfakis, Joao HF Pedra

**Affiliations:** 1 Center for Disease Vector Research and Department of Entomology, University of California-Riverside, Riverside, CA, 92521, USA; 2 Department of Pathology, Center for Biodefense and Emerging Infectious Diseases and Center for Tropical Diseases, School of Medicine, University of Texas Medical Branch, Galveston, TX, 77555, USA; 3 Department of Medical Sciences, Quinnipiac University, Hamden, CT, 06518, USA; 4 Institute of Parasitology, Biology Centre, Academy of Sciences of the Czech Republic, Ceske Budejovice, Czech Republic

**Keywords:** Tick, *Ixodes scapularis*, Saliva, *Anaplasma phagocytophilum*, Rickettsial agent

## Abstract

**Background:**

*Ixodes scapularis* saliva enables the transmission of infectious agents to the mammalian host due to its immunomodulatory, anesthetic and anti-coagulant properties. However, how *I. scapularis* saliva influences host cytokine secretion in the presence of the obligate intracellular rickettsial pathogen *Anaplasma phagocytophilum* remains elusive.

**Methods:**

Bone marrow derived macrophages (BMDMs) were stimulated with pathogen associated molecular patterns (PAMPs) and *A. phagocytophilum.* Cytokine secretion was measured in the presence and absence of *I. scapularis* saliva. Human peripheral blood mononuclear cells (PBMCs) were also stimulated with Tumor Necrosis Factor (TNF)-α in the presence and absence of *I. scapularis* saliva and interleukin (IL)-8 was measured.

**Results:**

*I. scapularis* saliva inhibits inflammatory cytokine secretion by macrophages during stimulation of Toll-like (TLR) and Nod-like receptor (NLR) signaling pathways. The effect of *I. scapularis* saliva on immune cells is not restricted to murine macrophages because decreasing levels of interleukin (IL)-8 were observed after TNF-α stimulation of human peripheral blood mononuclear cells. *I. scapularis* saliva also mitigates pro-inflammatory cytokine response by murine macrophages during challenge with *A. phagocytophilum*.

**Conclusions:**

These findings suggest that *I. scapularis* may inhibit inflammatory cytokine secretion during rickettsial transmission at the vector-host interface.

## Background

Hematophagy occurred independently in more than 14,000 arthropod species and this adaptation required physiological, morphological, behavioral and biochemical changes [1,2]. Salivary gland secretion is among the most common physiological and biochemical adaptation in hematophagous arthropods and salivary proteins from ticks, mosquitoes, biting flies, fleas and other blood-feeding arthropods ensue defense against host homeostasis and inflammation [1-8]. Combating inflammation is particularly problematic for ixodid ticks because these arthropods have to feed for a prolonged period of time and are exposed to a wide range of immune cells [9,10]. Pioneering studies have characterized the physiology of tick salivary glands [11] and raised the importance of saliva as an instrumental force for immune evasion [12-15]. Several groups have demonstrated that both proteinaceous and non-proteinaceous components of tick saliva impair the complement system and the function of macrophages, dendritic, T and B cells [8,16-25]. From these studies it also became apparent that pathogens take advantage of the immunomodulatory properties of vector saliva to colonize the host.

Titus and Ribeiro were the first researchers to describe the role of arthropod salivary glands facilitating parasite transmission. In this seminal work, the authors showed that sandfly salivary glands facilitate *Leishmania* spp. transmission to the mammalian host [26]. Subsequent studies by many other groups demonstrated that the saliva of arthropod vectors potentiates the transmission of a wide-range of arthropod-borne pathogens, including bacteria, viruses and protozoal organisms. For example, in *Ixodes* spp., *Borrelia burgdorferi* outer surface protein C (OspC) binds to salivary protein 15 (Salp15), which then acts as a barrier to protect this spirochete against the host immune response [27]. Sialostatin L2, an *I. scapularis* cystatin protein also facilitates the growth of the Lyme disease agent *B. burgdorferi *[28]. In addition, *I. ricinus* saliva inhibits interferon and Toll-like receptor (TLR) signaling during cell stimulation with *B. afzelli *[29,30]. Finally, infection by the tick-borne encephalitis virus can be prevented by immunizing animals against a truncated recombinant form of a tick salivary protein named 64P [31].

*Anaplasma phagocytophilum* is a pathogen transmitted by ixodid ticks and causes human granulocytic anaplasmosis, an emerging infectious disease in the United States, Europe and Asia [32]. However, it remains mostly unknown whether tick saliva inhibits cytokine secretion by macrophages during stimulation with *A. phagocytophilum*. Macrophages were recently shown to be important for defense against *A. phagocytophilum* colonization [33]. In this study, we show that *I. scapularis* saliva inhibits extracellular and intracellular receptor signaling in both murine and human immune cells. We also demonstrate that tick saliva mitigates *A. phagocytophilum*-induced cytokine secretion by murine macrophages. This report expands previous scientific knowledge on the immunomodulatory properties of tick saliva and suggests that rickettsial agents may use *I. scapularis* saliva to inhibit inflammation at the vector-host interface.

## Methods

### Ethical statements

Blood samples were obtained from healthy, non-pregnant adults. This procedure was approved by the Human Research Review Board (HRRB number: HS-08-135) at the University of California-Riverside. All animal experiments were approved by the Institutional Animal Care and Use Committee (IACUC number: A-20110030BE) at the University of California-Riverside. We used C57BL/6 mice at 6–10 weeks of age purchased from Jackson Laboratories. Experimentation with *A. phagocytophilum* (HZ strain) was approved by the Biological Use Authorization Committee (BUA number: 20120020) at the University of California-Riverside. *A. phagocytophilum* was grown in HL-60 cells (ATCC CCL-240). HL-60 cells were maintained in Iscove’s Modified Dulbecco’s Media (IMDM) with L-glutamine and hydroxyethyl piperazineethanesulfonic acid (HEPES) (Thermo Scientific), 20% heat-inactivated fetal bovine serum (FBS) (Sigma) in 5% CO_2_ and humidified air at 37°C, as previously described [33].

### Reagents

Lipopolysaccharide (LPS), Pam3CSK4, Zymosan, *Porphyromonas gingivalis* (PG)-LPS and muramyl dipeptide (MDP) were obtained from Invivogen. DOTAP was obtained from Roche. Human recombinant TNF-α was purchased from R&D Systems.

### Cell isolation and tick saliva collection

The generation of bone marrow-derived macrophages (BMDMs) and tick saliva has been previously described [33-35]. Briefly, mouse femurs were flushed and bone marrow cells were differentiated in complete Dulbecco’s Modified Eagle Medium (DMEM) (Fisher) supplemented with 30% L929 cell-conditioned media, 10% FBS and 1% PSA (100 U/mL penicillin, 100 mg/ml streptomycin, and 0.25μg/ml amphotericin) (Fisher). Cells were cultured at 37°C in a 5% CO_2_ tissue culture incubator for 5–6 days, with media changed on day 3. Human peripheral blood mononuclear cells (PBMCs) were isolated by using the Polymorphprep protocol (Axis Shield).

We collected *I. scapularis* saliva 4–5 days after feeding because studies suggest that transmission of *A. phagocytophilum* initiates slowly between 24 and 48 hours and is enhanced during rapid feeding to repletion around 72 h–96 h post tick attachment [36-38]. Therefore, saliva from *I. scapularis* would reflect actual conditions during *A. phagocytophilum* transmission at the vector-host interface. In addition, *I. scapularis* saliva collection at 24–48 hours is technically very challenging. The alternative would be using salivary glands. However, salivary glands bring a technical artifact to the system because this organ in ticks is rich in intracellular proteins and other immune effectors such as nucleotides, which may skew cytokine response in immune cells. To isolate vector saliva*, I. scapularis* ticks were allowed to feed on New Zealand white rabbits. A restraining collar was placed around the neck of each rabbit, and their ears were covered prior to tick exposure. Ticks were permitted to engorge for 4–5 days on the ear of a rabbit. Upon harvesting, ticks were rinsed in distilled water and were immediately fixed to glass slides with double-sided tape. A sterile glass micropipette was placed around the hypostome to collect saliva. Salivation was induced by the application of pilocarpine to the scutum of the tick. Saliva was pooled and stored at −80°C for use.

### Immune cell stimulation

BMDMs from C57BL/6 mice were stimulated with the TLR agonists LPS (500 ng/ml), Pam3CSK4 (1 μg/ml), Zymosan (10 μg/ml) and PG*-*LPS (500 ng/ml), the Nod2 stimulant MDP (10 μg/ml) or *A. phagocytophilum* (multiplicity of infection (MOI) 10 and 50) at indicated dilutions of tick saliva. Human PBMCs were purified by using the Polymorphprep protocol (Axis Shield), plated for 2 hours and incubated with tick saliva for 30 minutes followed by 4 hours of stimulation with 100 ng/ml of Tumor Necrosis Factor (TNF)-α. Pro-inflammatory cytokines such as TNF-α, interleukin (IL)-12p40, IL-6 and IL-1β or the chemokine IL-8 (CXCL8) were measured by ELISA.

### ELISA

Mouse TNF-α, IL-1β, IL-6 and human IL-8 were measured with the BD OptEIA Set from BD Biosciences. Mouse IL-12p40 was measured with capture and detection antibodies from eBiosciences. For the ELISA assays, wells were coated with recommended capture antibody dilutions in freshly prepared coating buffer (0.1 M sodium carbonate, pH 9.5). Plates were sealed and incubated overnight at 4°C followed by aspiration. Wells were then washed 3 times with ≥ 300 μL/well of freshly prepared wash buffer (phosphate buffered saline (PBS) with 0.05% Tween-20). After the last wash, plates were inverted and blotted on absorbent paper to remove any residual buffer. Wells were then blocked with ≥ 200 μL/well of assay diluent (PBS with 10% FBS, pH 7.0) and incubated at room temperature for 1 hour. Plates were washed 3 times with wash buffer. Then, standards and sample dilutions were prepared in assay diluent, as recommended (BD Biosciences). 100 μL of samples and standards were pipetted into the wells, incubated for 2 hours at room temperature followed by 5 washes. 100 μL of detection antibodies were diluted in assay diluent and added to each well. Plates were sealed and incubated for 1 hour at room temperature. Plates were washed 5 times. Then, 100 μL of enzyme reagent (BD OptEIA Set from BD Biosciences) were diluted in assay diluent, pipetted into each well and incubated for 30 minutes at room temperature. Wells were aspirated and washed 7 times with wash buffer. 100 μL of substrate solution (BD OptEIA Set from BD Biosciences) were added to each well and incubated for 30 minutes (without plate sealer) at room temperature in the dark. 50 μL of 2 N H_2_SO_4_ was added to each well. Absorbance was read in the ELISA plate reader (Bio-Rad) at 450 nm within 30 minutes. Background was corrected by reading the subtract absorbance at 570 nm.

### Cell death assay

Cell death was assayed by measuring lactate dehydrogenase (LDH), as recommended by the manufacturer (Takara). Briefly, 100 μL of each sample was placed into a well. Then, a catalyst solution (Takara) was added to the samples and controls. Samples were incubated for 10–30 minutes, at room temperature, protected from light. Reactions were stopped at the end of the incubation period by adding 50 μL of 1 N HCl. Absorbance was measured at 490 nm. The iMark microplate absorbance reader (Bio-Rad) was used according to the manufacturer’s instruction.

### Statistical analysis

Data were expressed as means ± standard errors of the means (SEM). The following parametric analyses were used: unpaired Student’s *t* test (two-group comparisons); one-way analysis of variance (ANOVA) (comparisons of three or more groups); Bonferroni post hoc multiple-comparison tests were used following ANOVA. All statistical calculations were performed using GraphPad Prism version 5.04. Graphs were made using GraphPad Prism version 5.04. *P* < 0.05 was considered statistically significant.

## Results

### *I. scapularis* saliva diminishes inflammatory cytokine secretion by murine macrophages

**Figure 1:**
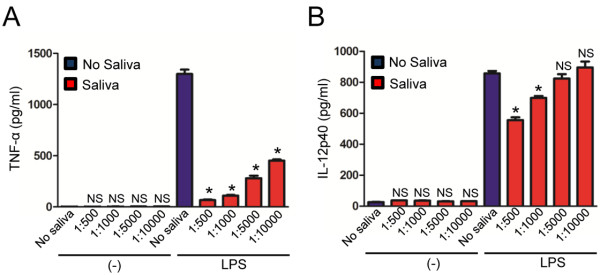
***I. scapularis *saliva mitigates LPS-mediated cytokine secretion by murine macrophages.** BMDMs (8×10^5^) from C57BL/6 mice were stimulated with LPS (500 ng/ml) for 18 hours, in the presence or absence of indicated dilutions of tick saliva. (**A**) TNF-α and (**B**) IL-12p40 were measured by ELISA. Tick saliva was added 2 hours before stimulation. Responses were measured in triplicate and presented as mean ± SEM within the representative experiment. Experiments were repeated three times. *P < .05, One-way ANOVA, post-hoc Bonferroni; (−) non-stimulated cells. NS – not significant.

**Figure 2:**
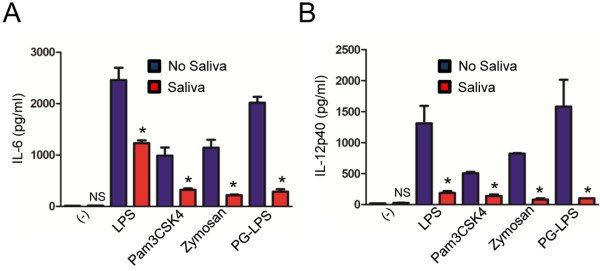
***I. scapularis *saliva inhibits TLR-mediated cytokine secretion by macrophages.** BMDMs (8×10^5^) from C57BL/6 mice were stimulated with LPS (500 ng/ml), Pam3CSK4 (1 μg/ml), Zymosan (10 μg/ml) and PG*-*LPS (500 ng/ml) for 18 hours in the presence or absence of tick saliva (1:500 dilution). Tick saliva was added 2 hours before stimulation. (**A**) IL-6 and (**B**) IL-12p40 were measured by ELISA. Responses were measured in triplicate and presented as mean ± SEM within the representative experiment. Experiments were repeated three times. *P < .05, Student’s t test. (−) non-stimulated cells. NS – not significant.

Tick saliva has immunomodulatory properties [1,2]. To determine whether tick saliva inhibits macrophage function, we first stimulated mouse BMDMs with LPS in the presence or absence of different dilutions of *I. scapularis* saliva. Cytokine levels were not altered during BMDM stimulation with tick saliva alone, suggesting that the saliva does not carry any contaminants, pathogen-associated molecular (PAMPs) or danger-associated molecular (DAMPs) patterns. As expected, LPS induced high levels of cytokine secretion in murine BMDMs (Figures 1 and 2). However, *I. scapularis* saliva inhibited secretion of both TNF-α and IL-12p40 by BMDMs after stimulation with LPS (Figure 1). This effect was more pronounced for TNF-α, as a tick saliva dilution of 1:10000 (v/v) still affected cytokine secretion by BMDMs during LPS stimulation (Figure 1A). A reduction in IL-12p40 secretion by macrophages after LPS stimulation was only observed for a tick saliva dilution of 1:1000 (v/v) and below (Figure 1B).

**Figure 3:**
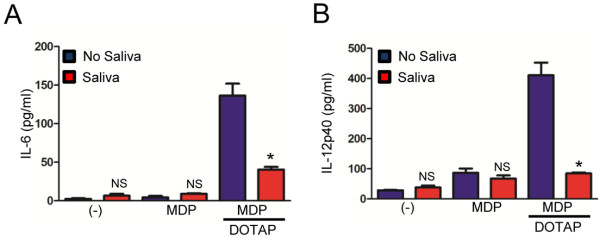
***I. scapularis *saliva impairs Nod2-mediated cytokine secretion by murine macrophages.** BMDMs (1×10^6^) from C57BL/6 mice were stimulated with MDP (10 μg/ml) or DOTAP (10 μg/ml) + MDP (10 μg/ml) for 20 hours, in the presence or absence of tick saliva (1:500). The secretion of (**A**) IL-6 and (**B**) IL-12p40 were measured by ELISA. Responses were measured in triplicate and presented as mean ± SEM within the representative experiment. Experiments were repeated three times. *P < .05, Student’s t test. (−) non-stimulated cells. NS – not significant.

We then stimulated mouse BMDMs with a wide range of TLR ligands and measured IL-6 and IL-12p40 secretion in the presence of *I. scapularis* saliva. Tick saliva 1:500 (v/v) inhibited IL-6 and IL-12p40 secretion by BMDMs when stimulated with TLR agonists, such as LPS, Pam3CSK4, Zymosan and PG-LPS (Figure 2). Next, we stimulated BMDMs with Nod1 and Nod2 agonists to determine whether the effect of *I. scapularis* saliva on cytokine secretion was restricted to TLRs. Nod1 and Nod2 are considered cytosolic receptors and are part of the Nod-like receptor (NLR) protein family [39]. The Nod1 and Nod2 agonists iE-DAP and MDP did not induce cytokine production in BMDMs during extracellular stimulation (Figure 3; *data not shown*). However, transfection of MDP to the cytosol using the cationic lipid DOTAP led to secretion of both IL-6 and IL-12p40 by BMDMs (Figure 3). Further, *I. scapularis* saliva inhibited IL-6 and IL-12p40 secretion mediated by the transfected Nod2 agonist MDP at the saliva dilution of 1:500 (v/v). Taken together, we report that *I. scapularis* saliva mitigates cytokine secretion by BMDMs during extracellular and cytosolic stimulation of TLR and NLR pathways.

### *I. scapularis* saliva inhibits chemokine secretion by human peripheral blood mononuclear cells

**Figure 4:**
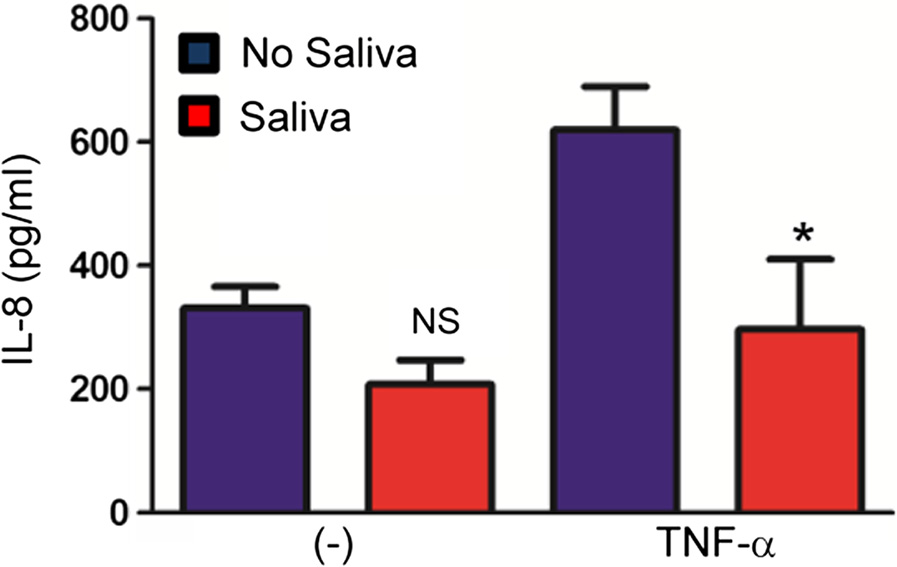
***I. scapularis *saliva decreases IL-8 secretion by human peripheral blood mononuclear cells.** PBMCs (9×10^5^) were purified and plated for 2 hours. Cells were incubated with tick saliva for 30 minutes followed by 4 hours of stimulation with 100 ng/ml TNF-α. The secretion of IL-8 (CXCL8) was measured by ELISA. Responses were measured in triplicate and presented as mean ± SEM within the representative experiment. *P < .05, Student’s t test. (−) non-stimulated cells. NS – not significant.

We then tested whether the effect observed for *I. scapularis* during cytokine secretion was restricted to murine BMDMs. We isolated human PBMCs from the blood of healthy and immunocompetent adults and stimulated these immune cells with the pro-inflammatory cytokine TNF-α. TNF-α has been extensively used to stimulate cytokine secretion in immune cells [40]. We included human PBMCs in our studies for two reasons. First, we would like to show that the inhibitory aspect of tick saliva is not only restricted to murine immune cells. To the contrary, it can also occur during stimulation of human immune cells. Second, PBMCs are a heterogenous group of immune cells. Therefore, we reasoned that PBMCs would be ideal to demonstrate the inhibitory cytokine capacity of tick saliva in a mixed group of immune cells. The chemokine IL-8 (CXCL-8) was used as a read-out in our analysis. PBMCs produced large amounts of IL-8 when stimulated with TNF-α (Figure 4). Similar to our findings observed in murine BMDMs, IL-8 (CXCL8) secretion by PBMCs stimulated with recombinant human TNF-α was diminished in the presence of tick saliva (Figure 4). Overall, our results show that *I. scapularis* saliva is able to mitigate cytokine and chemokine secretion by both murine and human immune cells.

### *I. scapularis* saliva lessens inflammatory cytokine secretion by murine macrophages during *A. phagocytophilum* stimulation

**Figure 5:**
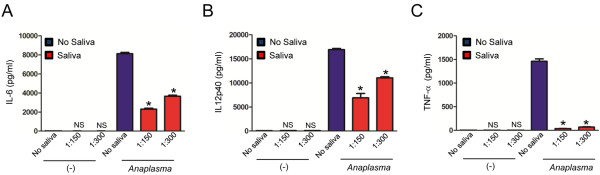
***I. scapularis *saliva mitigates cytokine secretion by macrophages during *A. phagocytophilum *stimulation in a dose-dependent manner.** BMDMs (1×10^6^) from C57BL/6 mice were stimulated with the wild-type *A. phagocytophilum* HZ strain (MOI 50) for 18 hours in the presence or absence of tick saliva (1:150 and 1:300 dilution). (**A**) IL-6, (**B**) IL-12p40 and (**C**) TNF-α were measured by ELISA. Responses were measured in triplicate and presented as mean ± SEM within the representative experiment. *P < .05, One-way ANOVA, post-hoc Bonferroni; (-) non-stimulated cells. NS – not significant.

**Figure 6:**
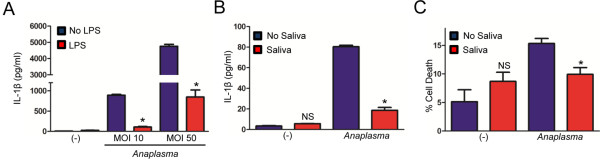
***I. scapularis *saliva decreases IL-1β secretion by macrophages during *A. phagocytophilum *stimulation.** (**A**) BMDMs (1×10^6^) from C57BL/6 mice were primed with LPS (500 ng/nl) for 24 hours, washed and then stimulated with the *A. phagocytophilum* HZ strain (MOIs 10 and 50) for another 24 hours. IL-1β was measured by ELISA. (**B**) BMDMs (1×10^6^) from C57BL/6 mice were stimulated with the wild-type *A. phagocytophilum* HZ strain (MOI 50) for 18 hours in the presence or absence of tick saliva (1:150 dilution). IL-1β was measured by ELISA. (**C**) Cell death was measured using the LDH assay. Responses were measured in triplicate and presented as mean ± SEM within the representative experiment. *P < .05, Student’s t test. (−) non-stimulated cells. NS – not significant.

To determine whether the effect of tick saliva was restricted to TLR and NLR ligands, we stimulated BMDMs with the *I. scapularis* rickettsial pathogen *A. phagocytophilum*. *A. phagocytophilum* induced the secretion of large amounts of cytokines by murine macrophages (Figures 5 and 6A-B). Nonetheless, tick saliva was also efficient in reducing cytokines by BMDMs, such as IL-6, IL-12p40 and TNF-α during *A. phagocytophilum* stimulation (Figure 5). Similar to BMDM stimulation with LPS, the effect of tick saliva was more pronounced on TNF-α secretion by BMDMs during pathogen stimulation. TNF-α secretion was completely abolished when BMDMs were stimulated with *A. phagocytophilum* in the presence of tick saliva (Figure 5C).

Overall, the inhibitory effect of tick saliva on cytokine secretion by BMDMs during *A. phagocytophilum* stimulation was best observed at a 1:150 dilution (v/v). Experiments performed with tick saliva at a 1:300 dilution (v/v) also showed reduction of cytokine secretion by BMDMs during *A. phagocytophilum* stimulation (Figure 5). However, the effect was milder (although statistically significant) for IL-6 and IL-12p40 (Figure 5A and B). We did not detect a dilution effect on TNF-α secretion by BMDMs at a 1:300 dilution (v/v) (Figure 5C). These results suggest that the inhibition threshold for tick saliva on TNF-α secretion by BMDMs during *A. phagocytophilum* stimulation is greater than 1:300 (v/v) compared to 1:150 dilution (v/v). It is unclear why more concentrated tick saliva is required to inhibit cytokine secretion during *A. phagocytophilum* stimulation when compared to individual TLR or NLR agonists. We reasoned that the presence of multiple PAMPs in a pathogen, such as *A. phagocytophilum*, may require a stronger dose of tick saliva to mitigate cytokine secretion by BMDMs.

### *I. scapularis* saliva inhibits IL-1β secretion by murine macrophages during *A. phagocytophilum* stimulation

A dogma has emerged in the last few years in which the production and release of IL-1β are the result of a two-tier system: one signal is induced by pattern recognition receptors or pro-inflammatory cytokines, which activates the transcription and translation of IL-1β via the transcription factor nuclear factor (NF)-κB. This signal is also referred to as priming and it is typically done by LPS stimulation of immune cells. The second signal is mediated by the inflammasome, a protein scaffold that cleaves IL-1β into its mature form through caspase-1 activation [41-43]. Our studies show that IL-1β secretion triggered by *A. phagocytophilum* during BMDM stimulation does not require LPS priming. To the contrary, LPS priming in BMDMs before *A. phagocytophilum* stimulation inhibited IL-1β secretion in a dose-dependent manner (Figure 6A). These results are consistent with the lack of genes for LPS and peptidoglycan synthesis in the *A. phagocytophilum* genome [44] and suggest that molecules other than LPS may prime BMDMs for the production of pro-IL-1β. Further, tick saliva inhibited *A. phagocytophilum*-induced IL-1β secretion by BMDMs (Figure 6B). IL-1β secretion may be coupled to a cell death phenomenon named pyroptosis [45]. We did not detect high levels of cell death during *A. phagocytophilum* stimulation of BMDMs in the absence of tick saliva (~10-15%) (Figure 6C). However, this is not entirely surprising, as *A. phagocytophilum* does not cause much cell death during infection [32,46]. Based on these findings, we conclude that in the *A. phagocytophilum* model, cytokine secretion is a better indicator of the anti-inflammatory features of tick saliva when compared to cell death.

## Discussion

Inflammation is characterized by complex interactions between innate and adaptive immunity [47]. Pro-inflammatory cytokines and chemokines recruit immune cells to the site of tick feeding. Tick salivary proteins then mitigate the secretion of cytokines by immune cells, thereby, diminishing inflammation [1,2,4]. Despite significant progress in the past decades, how ectoparasites, such as ticks, regulate host innate immune signaling during transmission of the rickettsial agent *A. phagocytophilum* to the mammalian host remains mostly elusive. In this study, we demonstrate that *I. scapularis* saliva has the ability to inhibit cytokine secretion by murine and human immune cells. These findings are supported by our results showing that extracellular and cytosolic stimulation of macrophages with PAMPs can be inhibited by *I. scapularis* saliva. We also performed experiments with *A. phagocytophilum* and demonstrated that similar mitigation effects occur in macrophages. The implications for these findings are wide in scope as ticks, mosquitoes, biting flies, fleas and blood-feeding bugs have also evolved similar strategies for modulating host defenses [2].

We reported an effect of tick saliva on TLR and NLR signaling in macrophages. Although the effect of tick saliva was previously demonstrated during TLR stimulation of dendritic cells [29,30,48-50], whether *I. scapularis* saliva affects the response to stimulation of murine macrophages and human peripheral blood mononuclear cells had not been determined. Macrophages and peripheral blood mononuclear cells are important because these immune cells respond to *A. phagocytophilum* infection [33,51]. To our knowledge, we describe for the first time that secretion of IL-6 and IL-12p40 after stimulation with the Nod2 ligand MDP was diminished in macrophages during treatment with tick saliva. Nod2 has emerged as a critical regulator for immunity and inflammation since it activates canonical and non-canonical NF-κB signaling, mitogen-activated protein kinases, cytokines, chemokines and antimicrobial reactive oxygen species [39].

Previously, we showed that *A. phagocytophilum* is partially recognized by the NLRC4 inflammasome [52], a protein scaffold that regulates the secretion of IL-1β and IL-18 [45]*.* We also demonstrated that mice deficient in *caspase-1* and *asc*, essential components of the inflammasome, were more susceptible than wild-type animals to *A. phagocytophilum* infection. These findings were due to the absence of IL-18 secretion and reduced interferon (IFN)-γ levels in the peripheral blood. It is unclear how *I. scapularis* saliva regulates IL-1β secretion by macrophages during *A. phagocytophilum* stimulation. However, it is possible that multiple salivary proteins regulate IL-1β secretion during hematophagy. Ticks have large genomes and carry many gene paralogs [53]. These gene paralogs may act redundantly to provide inhibition of immune protein scaffolds in the mammalian host. Two earlier articles provided experimental support for this hypothesis. Ramachandra and Wikel showed that salivary gland extracts from the tick *Dermacentor andersoni* reduced IL-1 levels during the early phases of tick feeding [54], whereas Fuchsberger *et al*., 1995 determined that human IL-1β secretion was mitigated when treated with LPS and salivary gland extracts from partially fed adult female *Rhipicephalus appendiculatus*[55].

In addition, *A. phagocytophilum* may need redundant mechanisms of innate immune recognition to trigger IL-1β secretion. Secretion of IL-1β requires NF-κB activation to generate pro-IL-1β [56,57]. Dumler and colleagues demonstrated that *A. phagocytophilum* triggers TLR2 activation during immune cell stimulation [58]. TLR activation is known to initiate NF-κB signaling in immune cells [59]. More recently, our group participated in a study showing that receptor interacting protein-2 (RIP2) affects *A. phagocytophilum* infection in mice [60]. RIP2 is an adaptor molecule for the innate immune receptors Nod1/2, which also regulates NF-κB signaling [61]. Finally, assembly of a multi-protein complex coined “inflammasome” is critical for IL-1β secretion [56,57]. We previously demonstrated that the inflammasome is critical for immunity against *A. phagocytophilum* infection [52]. Taken together, our findings reinforce the notion that *A. phagocytophilum* immunity is multi-factorial, and suggests a holistic inhibitory effect of tick saliva on innate immunity. This is important because a pathogen such as *A. phagocytophilum* may need multiple layers of immune evasion during transmission. Therefore, the holistic properties of tick saliva may be a major strategy of host immune evasion during pathogen transmission.

The above hypothesis is supported by several lines of evidence. Post-genomic approaches show that *A. phagocytophilum* actively modulates gene expression in ticks [62,63]. Recently, the P11 salivary protein was shown to be required for *A. phagocytophilum* migration from hemocytes to the salivary glands in ticks [64]. Another salivary gland protein named SALP16 was deemed important for *A. phagocytophilum* survival within the tick vector [65]. *A. phagocytophilum* alters the monomeric/filamentous (G/F) actin ratio leading to the translocation of phosphorylated/G-actin to the nucleus [66]. This event affects *salp16* gene transcription in association with the RNA polymerase II (RNAPII) and the TATA-binding protein. Fikrig and colleagues have also demonstrated that *A. phagocytophilum* appears to increase the ability of *I. scapularis* to survive in cold temperatures by up-regulating an antifreeze glycoprotein [67] and α1, 3- fucosyltransferases, which are important for pathogen colonization [68]. On the other hand, the Janus kinase (JAK)-signaling transducer activator of transcription (STAT) pathway seems to be important for the restriction of *A. phagocytophilum* infection in ticks. Clearly, further studies are necessary to determine the contribution of salivary proteins to *A. phagocytophilum* pathogenesis and immunity.

Elucidating the underlying effect of tick saliva *in vivo* should be considered a high-priority in tick research. Several experiments show that findings obtained *in vitro* may sometimes differ from those occurring *in vivo*, and mRNA and protein levels do not necessarily correlate well [1]. *In vivo* characterization of tick salivary proteins, however, is not a trivial task. The presence of multiple paralogues in the *I. scapularis* genome [53] make the use of siRNA or dsRNA technology challenging because of known off-target effects [69]. Another technical limitation is the lack of strategies to introduce and/or delete genes in ticks. Thereby, we were unable to generate knock-out, knock-in or transgenic *I. scapularis*. The development of this technology would enable researchers to characterize tick salivary proteins and clarify underlying events at the vector-pathogen-host interface. Despite all these technical restrictions, we posit that *I. scapularis* saliva may inhibit cytokine secretion during rickettsial transmission. We look forward with reasonable confidence that our findings may be used as a prelude for future *in-vivo* experimentation.

## Conclusions

To facilitate blood-feeding habits and counteract homeostasis and inflammation in the host, arthropod vectors have evolved a sophisticated pharmacological portfolio. Our studies add to a continuum of research over the past 20–30 years and show that *I. scapularis* saliva diminishes cytokine secretion by mouse and human immune cells during TLR and NLR stimulation. We also show that cytokine response by macrophages is diminished in the presence of *I. scapularis* saliva during *A. phagocytophilum* stimulation. Taken together, our findings should pave the ground for new research directions in the field of *A. phagocytophilum* pathogenesis and immunity and may have direct implications for understanding how ticks circumvent defenses promoted by the mammalian immune system.

## Abbreviations

TLRs: Toll-like receptors; NLRs: Nod-like receptors; NF-κB: Nuclear factor; IL: Interleukin; TNF-α: Tumor Necrosis Factor; PAMPs: Pathogen Associated Molecular Patterns; LPS: Lipopolysaccharide; MDP: Muramyl dipeptide; BMDMs: Bone marrow-derived macrophages; PBMCs: Peripheral blood mononuclear cells.

## Competing interests

The authors declare that they have no competing interests.

## Authors’ contributions

GC, MSS, MS and OSS carried out the experimental work. SKW and MK analyzed the data and provided intellectual support. JHFP directed the project and wrote the manuscript. All authors read and approved the final manuscript.
